# Simultaneous determination of four cobalamins in rat plasma using online solid phase extraction coupled to high performance liquid chromatography-tandem mass spectrometry: Application to pentylenetetrazole-induced seizures in Sprague-Dawley rats

**DOI:** 10.1371/journal.pone.0269645

**Published:** 2022-06-07

**Authors:** Xiaomei Jiang, Yan Wang, Jia Liu

**Affiliations:** 1 Department of Precision Instrument and Opto-Electronics Engineering, Tianjin University, Tianjin, China; 2 Department of Microbiology and Infectious Disease Center, School of Basic Medical Sciences, Peking University Health Science Center, Beijing, China; Fisheries and Oceans Canada, CANADA

## Abstract

A high throughput and high sensitive online solid phase extraction coupled to high performance liquid chromatography-tandem mass spectrometry method was established and validated for simultaneous quantification of four cobalamins including OHCbl, CNCbl, AdoCbl and MeCbl in rat plasma. Analytes were first enriched on an online SPE Shim-pack MAYI-ODS column and then separated using a Poroshell 120 EC C18 analytical column. The linear range was from 0.05 to 5 ng/ml and the LLOQ was 0.05 ng/ml for all four cobalamins. In addition, this method was applied to determine the cobalamins levels in pentylenetetrazole-induced kindling rat model for the first time.

## 1. Introduction

**Fig 1:**
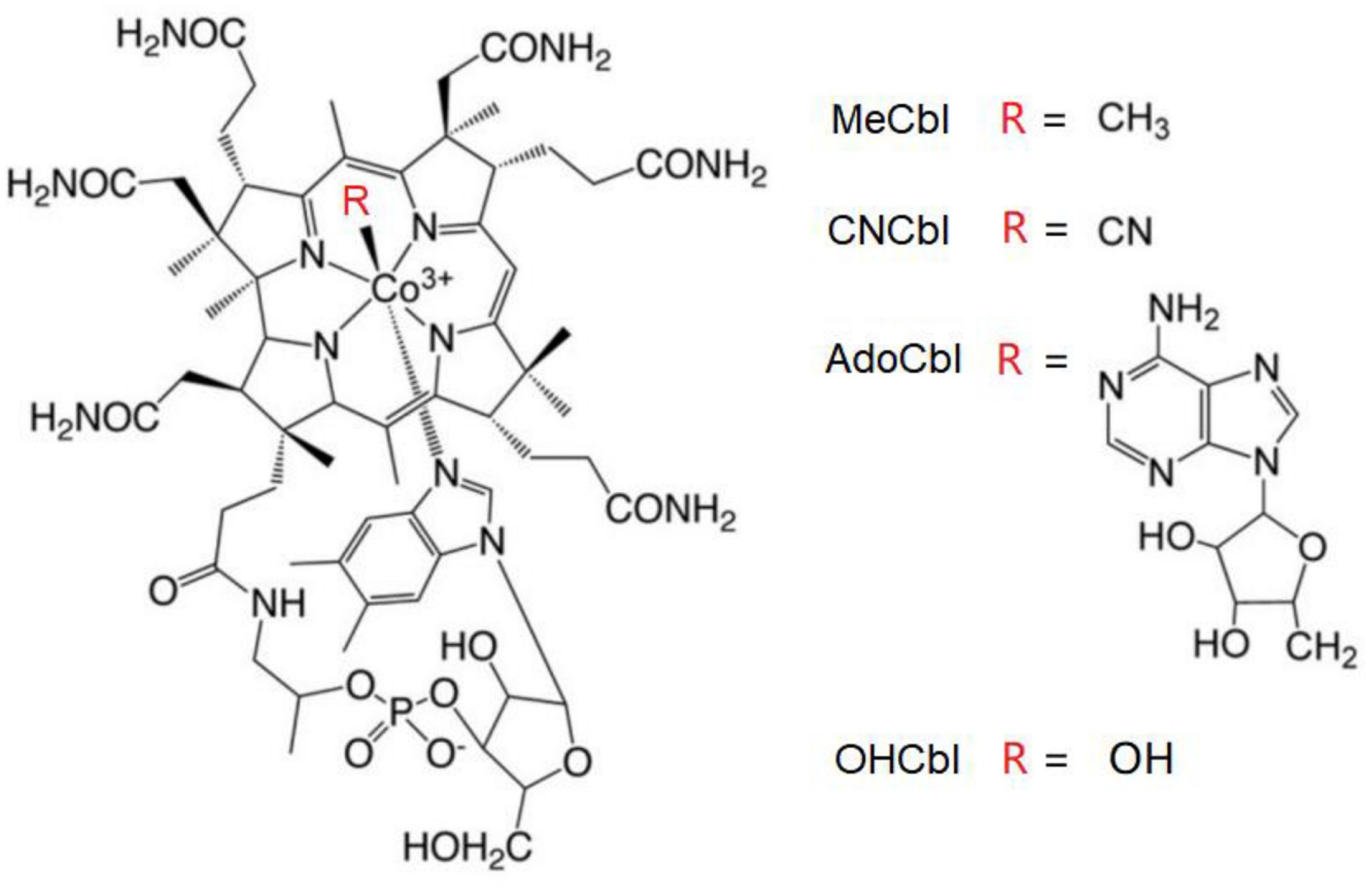
Chemical structures of four cobalamins. R group may be: OH (hydroxycobalamin, OHCbl), CN (cyanocobalamin, CNCbl), 5’-deoxyadenosyl (adenosylcobalamin, AdoCbl) and CH3 (methylcobalamin, MeCbl).

Cobalamins, also known as vitamin B12, are a group of water-soluble molecules which is an essential nutrient for all living organisms [1]. Vitamin B12 (cobalamins) has a complex chemical structure containing a central cobalt ion complexed with several organic ligands [2]. A Corrin ring binds with four binding sites of the cobalt ion, and the fifth site is occupied by a dimethylbenzimidazole group to form the basic skeleton of vitamin B12. The sixth site of the cobalt ion is occupied by cyano (cyanocobalamin, CNCbl), hydroxyl (hydroxycobalamin, OHCbl), methyl (methylcobalamin, MeCbl), or 5’-deoxyadenosyl groups (adenosylcobalamin, AdoCbl), shown in Fig 1.

Cobalamin is required to maintain many biological functions in human body. In human plasma, both the contents and functions of these four cobalamins are different. AdoCbl and MeCbl are biochemically active forms, while OHCbl and CNCbl are usually used as drugs or supplements [3]. OHCbl and CNCbl could not play their physiological roles until they are converted to AdoCbl or MeCbl. MeCbl is an essential factor in conversion of homocysteine to methionine, and AdoCbl is necessary in the conversion of succinyl coenzyme A, which is involved in Krebs cycle [4]. Cobalamins cannot be synthesized by the human body, and it must be obtained from foods or supplements [5]. Insufficient intake of vitamin B12 may lead to many medical complications such as pernicious anemia [6] and neurological disorders [7]. In order to evaluate the levels of active forms (AdoCbl and MeCbl) and to monitor the concentrations of OHCbl and CNCbl in therapeutic process, a rapid, sensitive, reliable and robust instrument method for analysis of cobalamins in biological samples especially in plasma is urgently needed.

Süleyman Bodur et al. analyzed cobalamin and cobalt in kefir samples using HPLC-UV and HPLC-ICP-OES [8]. B12 in biological fluid could be measured by HPLC-UV [9, 10], however the sensitivity was not high enough. Hong You group detected these four active vitamin B12s in dietary supplements by reversed-phase HPLC-DAD (Diode-Array Detection), however, detection and quantification limits were also relative high because of the low sensitivity of DAD instrument [11]. Although several MS-based methods were established to detect this vitamin in real samples [12, 13], they could only detect CNCbl, ignoring other kinds of cobalamins.

As far as we know, there is no high sensitive HPLC-MS/MS method exists for simultaneous determination of OHCbl, CNCbl, MeCbl and AdoCbl in biological samples. We here reported a high throughput online SPE-HPLC-MS/MS method in a single-step for detection of these four cobalamins in rat plasma. And this method was successfully applied to detect the four cobalamins in PTZ-induced kindling rat model for the first time.

## 2. Materials and methods

### 2.1. Chemicals and reagents

Optima LC-MS grade chemicals (ammonium formate, formic acid and acetic acid) and HPLC grade solvents (methanol and acetonitrile) were obtained from Fisher Scientific (Fairlawn, NJ, U.S.A.). Zinc sulfate heptahydrate (ZnSO_4_·7H_2_O) and pentylenetetrazole (PTZ) was obtained from Sigma-Aldrich Chemicals (St. Louis, MO, U.S.A.). Deionized water was purchased from Quchenshi Company (Guangdong, China). Sterilized saline was from Double-Crane Pharmaceutical Co., Ltd. (Beijing, China). CNCbl (purity: 98%) and MeCbl (purity: 98%) were purchased from Toronto Research Chemicals (Ontario, Canada). OHCbl (purity: ≥ 98%) and AdoCbl (purity: ≥ 98%) were purchased from Macklin (Shanghai, China). ^13^C_7_-Cyanocobalamin (CNCbl-^13^C_7_, 1μg/mL in methanol, isotopic purity: 99%) was used as internal standard (IS) and obtained from Cambridge Isotope Laboratories (MA, U.S.A.).

### 2.2. Animals

Institutional Animal Care and Use Committee of Beijing Vital River Laboratory Animal Technology Co., Ltd approved all animal experimental procedures with the protocol number of P2021064. 16 male adult Sprague-Dawley (SD) rats (weighing 240 ~ 300 g), obtained from Charles River Laboratories (Beijing, China), were used in this study. The animals were housed under controlled environmental conditions (ambient temperature 23 ± 2 °C, air humidity 50~70%, artificial 12 h dark/12 h light cycles) for at least 3 days before they were used for the experiments. Commercial rat pellets and water were freely available. All experiments were done at the same time in the morning to avoid the bias of circadian rhythms.

Rats were randomly divided into control (n = 8) and PTZ group (n = 8). PTZ groups received PTZ (20 mg/mL in saline) administration intraperitoneally once every 48 hrs. (40 mg/kg body weight) for 5 times, while the control rats were injected with an equal dose of saline. After each PTZ administration, all animals were observed for 30 min in individual cages. Their responses were recorded according to the following scale provided by W Fischer and H Kittner [14]: 0: no response; 0.5: weak nodding; 1: ear, face and eyelid twitching; 1.5: mild forelimb clonus; 2: myoclonic convulsions without rearing; 2.5: partial rearing and rapid clonic seizures of fore-limb; 3: myoclonic convulsions with rearing; 3.5: rearing and falling with intense bilateral fore-limb clonus; 4: clonic convulsion with loss of posture; 4.5: generalized clonic-tonic seizures with failure of righting reflex; 5: generalized clonic-tonic seizures and status epilepticus (≥ 2 min).

The rats were sacrificed 30 minutes after the last injection by euthanasia with carbon dioxide. Blood samples were obtained from the abdominal vein using 2.0 mL EDTAK2 coated micro centrifuge tubes, and plasma was isolated by centrifugation at 12000 g for 10 min. All plasma samples were stored at −80 °C until analysis.

### 2.3. Sample processing

Plasma samples were left to thaw at 4 °C, and then 100 μL plasma was transferred to an Eppendorf tube to which 10 μL IS working solution (20 ng/mL CNCbl-^13^C_7_ in methanol) was added. Proteins were then precipitated by adding 100 μL cold acetonitrile/methanol (3:1, v/v) and vortexing for 2 min. To precipitate sufficiently, 100 μL 0.2 M Zinc sulfate was further added and mixed by vortexing. After 10 minutes’ standing at -20 °C, samples were centrifuged at 16 000 g for 10 min. 200 μL supernatant was transferred to a 96-well plate for injection into the LC-MS system. All operations were conducted in the subdued light strictly.

### 2.4. Instrumentation

#### Online solid phase extraction (SPE) and chromatographic conditions

**Fig 2:**
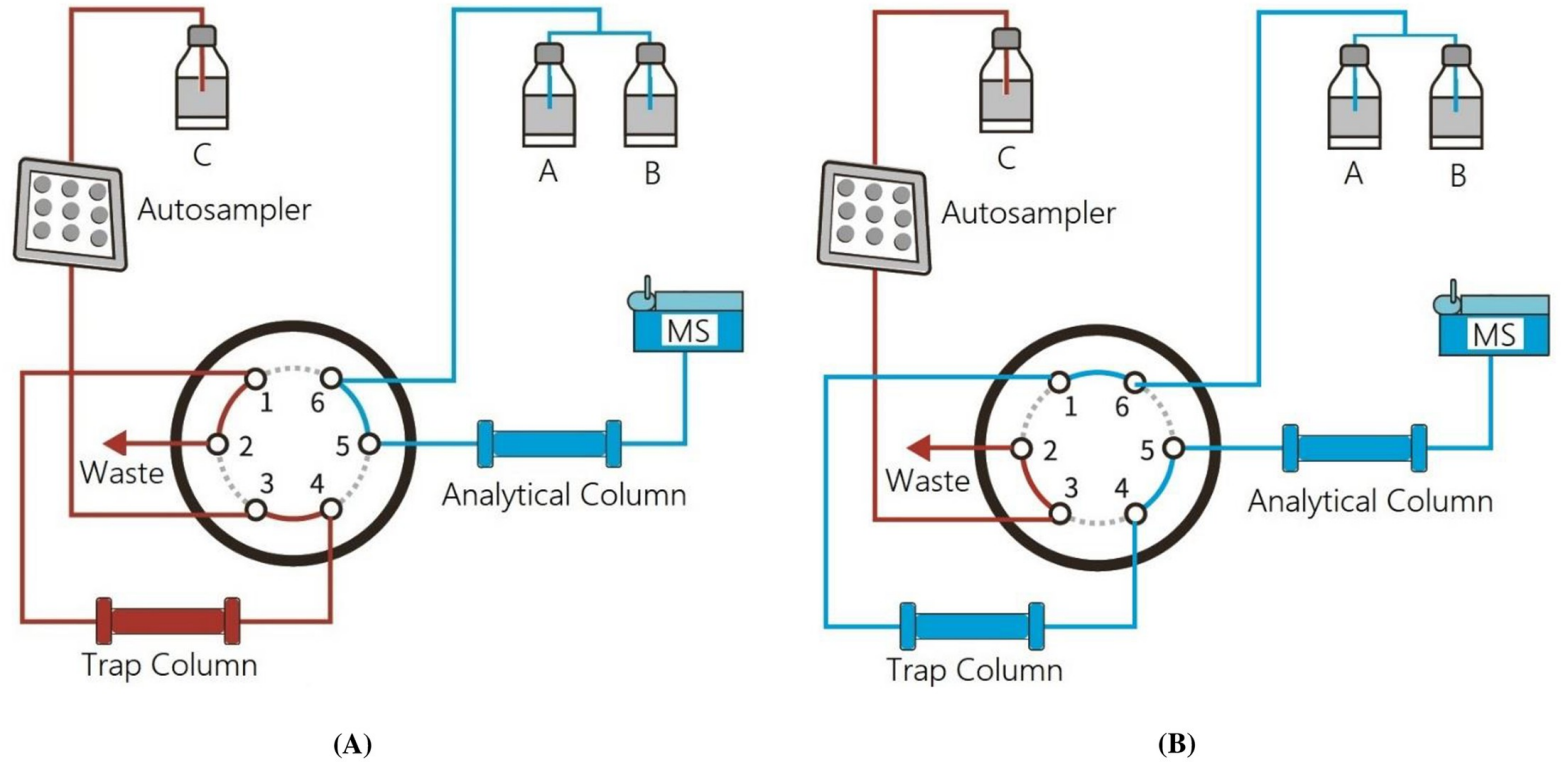
Schematic diagram of the online SPE and chromatographic separation system: (A) valve position I at loading samples, (B) valve position II at separating samples.

Fig 2 shows the Schematic diagram of the home-made online SPE and chromatographic separation system. The online SPE and chromatographic separation was carried out on a Shimadzu Prominence LC system equipped with a CMB20A controller, a SIL20AC auto-sampler, three LC20AD pumps and a CTO20AC column oven with a six-port switching valve in it (Shimadzu, Kyoto, Japan).

Online SPE was performed using a Shim-pack MAYI-ODS column (4.6 × 30 mm, 50 μm, Shimadzu, Kyoto, Japan) as the trap column. The prepared samples were kept at 5 °C in the auto-sampler compartment before injected. 50 μL of each sample was loaded onto the trap column using the isocratic pump delivering mobile phase C (5 mM ammonium formate with 0.1% (v/v) formic acid in water/methanol (95:5, v/v)) at a flow rate of 2 mL/min, as shown in Fig 2A. The analytes of interest were trapped on the trap column and other impurities/interferences were eluted to waste.

Chromatographic separation was performed using a Poroshell 120 EC C18 column (50 × 4.6 mm, 2.7 μm, Agilent, CA, USA) through a binary pump delivering 0.02% (v/v) acetic acid in water (pH 3.6) (mobile phase A) and methanol (mobile phase B). At 1.5 min, valve was switched to allow the binary pump carrying mobile phase A/B to back flush the analytes onto the analytical column at 0.7 mL/min (Fig 2B). The trap column and analytical column are both positioned in the column oven at 35 °C. The final mobile phase gradient and valve switching program are summarized in Table 1.

**Table 1:** The online SPE-HPLC-MS/MS gradient elution and valve switching program.

Isocratic pump Mobile Phase C		Binary pump			Valve switching program
Time (min)	Mobile Phase C Flow rate (mLmin-1)	Time (min)	Mobile Phase A (%)	Mobile Phase B (%)	Valve position
0	2	0	90	10	I
1.5	2	2	90	10	II
1.51	0	3	30	70	
6	0	4.5	30	70	I
6.01	2	4.6	0	100	
		6	0	100	
		6.01	90	10	
		8	90	10	

#### Mass spectrometry conditions

Mass spectrometric analysis was carried out in positive electrospray ionization mode (ESI+) using multiple reaction monitoring (MRM) scan type on a 4000 Q TRAP LC/MS/MS System (Applied Biosystems, MDS Sciex, Toronto, Canada). Nitrogen produced by a nitrogen generator (Airtech, Tokyo, Japan) was used as the de-solvation gas and collision gas, and the following general source conditions were used: ion-spray voltage (IS), 5500V; source temperature (TEM), 650 °C; Ion source gas 1 (GS1), 55 psi; ion source gas 2 (GS2), 50 psi; curtain gas (CUR), 30 psi; Collision gas (CAD), high. The MRM parameters, including declustering potential (DP), collision energy (CE), and collision cell exit-potential (CXP), were determined via direct infusion of each analyte and IS. Two transitions were selected for identification for each analyte and only one fragment ion was selected for IS. Compound specific parameters for MRM analysis are detailed in Table 2. The instruments were controlled by Analyst software version 1.6.3.

**Table 2:** Specific MRM parameters and retention times (RT) for each analyte and internal standard (IS).

Analyte	MRM transition	RT (min)	DP	CE	CXP
OHCbl	665.3 → 636.5^a^	3.60	80	25	14
	665.3 →147.2^b^	3.60	80	60	8
CNCbl	678.3 → 147.3^a^	3.80	85	60	8
	678.3 → 359.2^b^	3.80	85	32	15
AdoCbl	790.6 → 665.5^a^	3.85	80	30	15
	790.6 → 147.3^b^	3.85	80	80	8
MeCbl	672.8 → 665.4^a^	3.98	75	25	16
	672.8 → 147.1^b^	3.98	75	66	6
(IS)	682.0 → 154.2	3.80	85	60	8

^a^: quantifier transition,

^b^: qualifier transition.

### 2.5. Method validation

The established method was validated according to the Food and Drug Administration (FDA) guidance for industry on bioanalytical methods in terms of selectivity, linearity, lower limit of quantitation (LLOQ), precisions and accuracy, carryover effect and stability.

#### Preparation of stock solutions, internal standard working solution, calibrators and quality control samples

Stock solutions of OHCbl, CNCbl, MeCbl and AdoCbl were prepared in methanol at 1 mg/mL concentration individually and further diluted to 10 μg/mL in methanol, getting individual working standard solutions of each cobalamin. Then the individual working standard solutions were used to prepare the mixed stock solution of four cobalamins at 1 μg/mL in methanol, and aliquots of the mixed stock solution (100 μL) were stored at -80 °C. All solutions were also prepared in amber-glass bottles to protect vitamins from light-induced oxidation.

The internal standard (IS) working solution was prepared by diluting the CNCbl-^13^C_7_ solution (1 μg/mL in methanol) to a concentration of 20 ng/mL also in methanol.

Calibrators were prepared from the mixed stock solution at the following seven concentrations: 0.05, 0.10, 0.20, 0.50, 1.00, 2.00 and 5.00 ng/mL in water/methanol (1:1, v/v).

Quality control samples (QCs) were prepared by spiking the mixed stock solution into a pooled plasma sample from at least six different subjects at three levels: low-level (0.2 ng/mL), mid-level (1.0 ng/mL) and high-level (4.0 ng/mL). All of the calibrators and QCs were aliquoted and stored at -80 °C.

Like many other vitamins, MeCbl, AdoCbl and CNCbl is light-sensitive and could degrade to OHCbl by losing the corresponding ligand [3, 15]. Working with amber vials in subdued lighting is necessary to prevent such degradation. Stock solutions of CNCbl and CNCbl-^13^C_7_ were prepared in water and stored at 4 °C in amber vials for no longer than one month.

#### Selectivity

The FDA guideline defined selectivity as the capability of an analytical method to quantify and verify the targeted analytes in the presence of interfering components from matrix [16]. The selectivity of our method was studied by injecting blank (no analyte, no IS) and zero (blank plus IS) calibrators and single analyte while monitoring all MRM transitions under consideration. Significant signals from interference should not be detected at the corresponding retention time of the chosen compounds.

#### Linearity and LLOQ

Seven calibrators were prepared and injected to construct calibration curves determined by plotting the peak area ratios of analytes versus IS (y) and the theoretical concentrations of the calibrators (x) with “1/x^2^” weighting. Linearity was assessed using the correlation coefficient which was required to be > 0.99 over the 3 days.

The LLOQ was defined as the lowest analytical concentration of the calibration curve at which signal-to-noise ratio (S/N) ≥ 10 and accuracy and precision ≤ 20%.

#### Precision and accuracy

Precision was evaluated by measuring QCs in five independent series during the same day (intra-day precision) and over 3 consecutive days (inter-day precision). The coefficient of variance (CV) for replicate assays was calculated as the assessment of precision and should not exceed 15%.

Accuracy was determined by comparing the measured concentration of each QC to its theoretical concentration. Accuracy was expressed as % deviation (%dev) and should be ranged within ± 15%.

#### Carryover effect

Carryover was assessed with a blank of methanol run directly after injection of the highest standard in the calibration curve and was accepted if the response was ≤ 20% of the average response of LLOQ.

#### Stability

Rat plasms samples fortified with the four cobalamins at three concentration levels were analyzed for stability study. The stability of the four cobalamins under various stress conditions was evaluated: 3 cycles of freeze thaw, standing on a bench top at room temperature for 4 hours, standing in an auto-sampler after preparation at 5 °C for 24 hours, and long-term storage at -80 °C for 30 days.

## 3. Results and discussion

### 3.1. Optimization of sample pretreatment

Protein precipitation was used in sample pretreatment because of its simplicity and high-throughput. In order to obtain high recovery and appropriate retention, we tried several precipitants and various precipitation processes. 10% or 15% trichloroacetic acid (TCA) was first exploited as an acidic precipitant, however, the MS responses of these cobalamins were sacrificed especially for MeCbl. Organic precipitants could achieve a rapid and highly efficient precipitation, but the retention ability was lost for OHCbl, leading to more interferences and lower sensitivity.

Based on the preliminary experiments, the optimal precipitation process was obtained. Proteins were first precipitated by adding 100 μL cold acetonitrile/methanol (3:1, v/v) and vortexing for 2 min, and then 100 μL 0.2 M Zinc sulfate was further added to the mixture to precipitate sufficiently and to dilute organic phase.

We also investigated the stability of these cobalamins in various lab environments. Working with amber vials in subdued lighting is necessary to prevent their photodegradation.

### 3.2. Liquid chromatography and mass spectrometry method development

In this study, a high throughput and sensitive online solid phase extraction coupled to high performance liquid chromatography-tandem mass spectrometry approach was developed to determine four cobalamins in rat plasma. ^13^C_7_-Cyanocobalamin (CNCbl-^13^C_7_) was selected as internal standard because of its high similarity in chemical structure and physical property compared to the four cobalamins.

**Fig 3:**
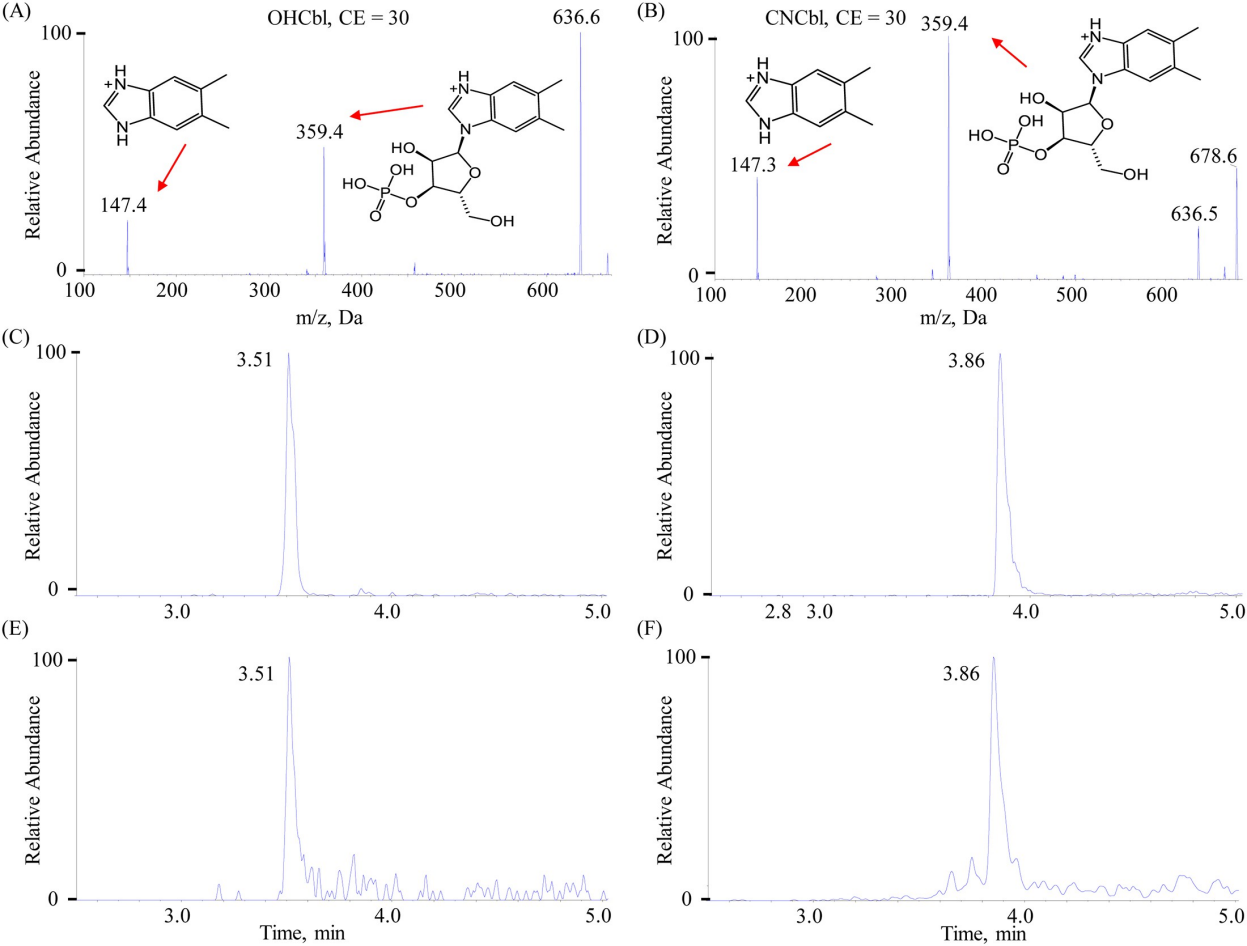
MS/MS spectra of (A) OHCbl and (B) CNCbl. Representative MRM chromatograms of (C) OHCbl and (D) CNCbl in standards. Representative MRM chromatograms of (E) OHCbl and (F) CNCbl in plasma.

Mass spectrometric parameters were optimized by infusing 5 μg/ml solutions of each cobalamin at a rate of 5.0 μL/min via a syringe pump. Fig 3A and 3B showcase the MS/MS spectra of OHCbl and CNCbl respectively. The fragment peaks were chemically attributed by the inserts. Two daughter ions were chosen for each cobalamin as the product ion in MRM scan mode as listed in Table 2: one was for quantification and the other was for qualification. The most suitable values of DP, EP, CE and CXP for each precursor-to-product transition were determined by monitoring the strongest MS response in MRM scan mode. The final optimized DP, EP, CE and CXP were detailed in Table 2. An 80 ms dwell time was used for each MRM transition in one single retention time window.

Cobalamins were trapped on the online SPE column and separated in the following liquid chromatography system. Three trap columns Shim-pack MAYI-ODS column (4.6 × 30 mm, 50 μm, Shimadzu, Kyoto, Japan), CAPCELL PAK MF Ph-1 S5 (4.0 × 20 mm, 5 μm, Daiso Chemical Co., Ltd., Japan) and CAPCELL PAK MF C8 (4.0 × 20 mm, 5 μm, Daiso Chemical Co., Ltd., Japan) were compared in the preliminary experiments. MAYI-ODS column exhibits the best retention performance for the four cobalamins, it was therefore selected in the following experiments. Two analytical columns HALO AQ-C18 Column (50 × 4.6 mm, 2.7 μm) and Poroshell 120 EC C18 column (50 × 4.6 mm, 2.7 μm, Agilent, CA, USA) were also tested, and Poroshell 120 EC C18 column was chosen for its good separation performance and lengthy working life.

After systematical optimization, sound separation and peak with symmetric shape and strong response was obtained for the four cobalamins. Fig 3C and 3D depict the representative MRM chromatograms of OHCbl and CNCbl in standards respectively.

### 3.3. Method validation

Four cobalamins were quantified by internal standard method to eliminate matrix effect and to minimize instrument fluctuation. Isotope analog CNCbl-^13^C_7_ was adopted as internal standard due to its high similarity to the four cobalamins in chemical-physico properties. Typical MRM chromatograms of OHCbl and CNCbl in plasma are demonstrated in Fig 3E and 3F respectively.

Method validation was conducted according to the Food and Drug Administration (FDA) guidance for industry on bioanalytical methods in terms of selectivity, linearity, lower limit of quantitation (LLOQ), precisions and accuracy, carryover effect and stability.

To assess specificity and selectivity, individual standards as well as IS were injected to LC-MS while monitoring all MRM transitions under consideration. After scrutinize all MRM transitions at the retention time of the chosen compounds, we found no interference peak appeared at the corresponding retention time.

Seven calibrators ranging from 0.05 ng/ml to 5 ng/ml were used to construct calibration curves of the four cobalamins. The peak area ratio of each cobalamin to IS was plotted against the calibrator concentration with “1/x^2^” weighting. The Linear equation and correlation coefficient (r) of each B12 were detailed in Table 3. All correlation coefficients were higher than 0.992, indicating the good linearity and reliability in quantification. Linear ranges were 0.05 ~ 5 ng/ml, and LLOQs were 0.05 ng/ml for all four cobalamins. LODs for MeCbl, AdoCbl and OHCbl were 0.01 ng/ml, and LOD for CNCbl was 0.02 ng/ml. Since cobalamins are endogenous, it is difficult to find an ideal blank matrix which should be rat plasma without these cobalamins. We thus chose water/methanol as a substitute of plasma matrix. The reliability of this matrix substitute was proved by the accuracies calculated from QC samples at three concentration levels, *vide infra*. We also compared the slope of the calibration curve in the matrix substitute to that prepared in pooled rat plasma, and our data revealed that it was acceptable to utilize water/methanol as a matrix substitute.

**Table 3:** Linear equation, correlation coefficient (r) and LOD of the four cobalamins.

Analyte	Linear equation	r	LOD (ng/ml)
MeCbl	y = 0.82253 x + 0.00803	0.99847	0.01
AdoCbl	y = 0.37173 x + 0.01943	0.99744	0.01
CNCbl	y = 0.41282 x—0.00516	0.9921	0.02
OHCbl	y = 0.11893 x + 0.01416	0.99448	0.01

QC samples at three concentration levels were injected repeatedly to evaluate the precisions and accuracies for intra-day (n = 6) and inter-day (n = 15). As displayed in Table 4, the intra-day precisions ranged from 1.83 to 11.9%, while inter-day precisions ranged from 4.68 to 13.9%, indicating decent precisions of our developed SPE-HPLC-MS/MS method. Accuracy was calculated dividing the theoretical concentration of QCs into that measured at low, middle and high concentrations. The intra-day accuracies ranged from 86.2 to 113%, and the inter-day accuracies 88.3 to 108%, which was tolerable according to the guidance.

**Table 4:** Method validation for SPE-HPLC-MS/MS analysis of four cobalamins: Precision and recovery values at different concentrations (0 ng/ml, 0.2 ng/ml, 1.0 ng/ml and 4.0 ng/ml) in quality control samples.

Analytes	Spiked (ng/ml)	Intraday (n = 6)		Interday (n = 15)	
		Accuracy (%)	RSD (%)	Accuracy (%)	RSD (%)
OHCbl	0^a^	-	11.9	-	8.45
	0.2	103	7.87	102	7.52
	1.0	108	7.80	95.8	12.4
	4.0	109	4.01	99.1	13.3
CNCbl	0^a^	-	5.32	-	10.9
	0.2	96.3	6.13	99.8	8.48
	1.0	86.2	4.18	87.2	5.07
	4.0	86.6	1.83	88.3	4.86
AdoCbl	0^a^	-	9.51	-	11.2
	0.2	89.8	9.31	103	13.9
	1.0	105	10.9	108	13.7
	4.0	107	10.6	105	10.8
MeCbl	0^a^	-	8.33	-	10.8
	0.2	113	8.84	108	9.26
	1.0	113	6.07	107	11.4
	4.0	111	4.60	104	9.38

^a^: No analytes were spiked into plasma samples. The concentration here refer to the endogenous concentration.

No carryover effect was observed when a blank sample ran directly after the highest concentration of calibrators.

Freeze-thaw stability, bench-top stability, auto-sampler stability and long-term stability of the four cobalamins were all assessed. The area under the peak of each cobalamin experienced various stress conditions was compared to that of the fresh prepared solution. Table 5 lists the recoveries of the four cobalamins in all conditions, which were between 88 to 114%. To be point out, though cobalamins were stable under these stresses, MeCbl, AdoCbl, CNCbl and CNCbl-^13^C_7_ were light sensitive, and working with amber vials in subdued lighting is necessary.

**Table 5:** Stability of four cobalamins in rat plasma in various stresses determined by SPE-HPLC-MS/MS (n = 3).

Analyte	Spiked (ng/ml)	Room temperature 4hr	3 freeze-thaw cycles	Auto-sampler 5°C 24hr	-80 °C 30 days
OHCbl	0.2	105%	95.4%	95.7%	94.2%
	1	94.6%	107%	111%	91.1%
	4	94.5%	91.0%	113%	90.9%
CNCbl	0.2	104%	103%	102%	95.5%
	1	107%	106%	109%	102%
	4	114%	103%	113%	94.7%
AdoCbl	0.2	98.7%	112%	106%	112%
	1	108%	114%	104%	105%
	4	112%	106%	112%	97.7%
MeCbl	0.2	108%	88%	94.3%	101%
	1	109%	106%	96.4%	98.4%
	4	112%	102%	104%	98.4%

### 3.4. Application of the method

Pentylenetetrazol (PTZ) could induce sprouting of the hippocampal mossy fibers during kindling progression with lower experimental animal mortality and is widely used as a convulsant in rat model [17, 18]. However, the B12 level in this PTZ-induced kindling rat model is still unknown.

**Fig 4:**
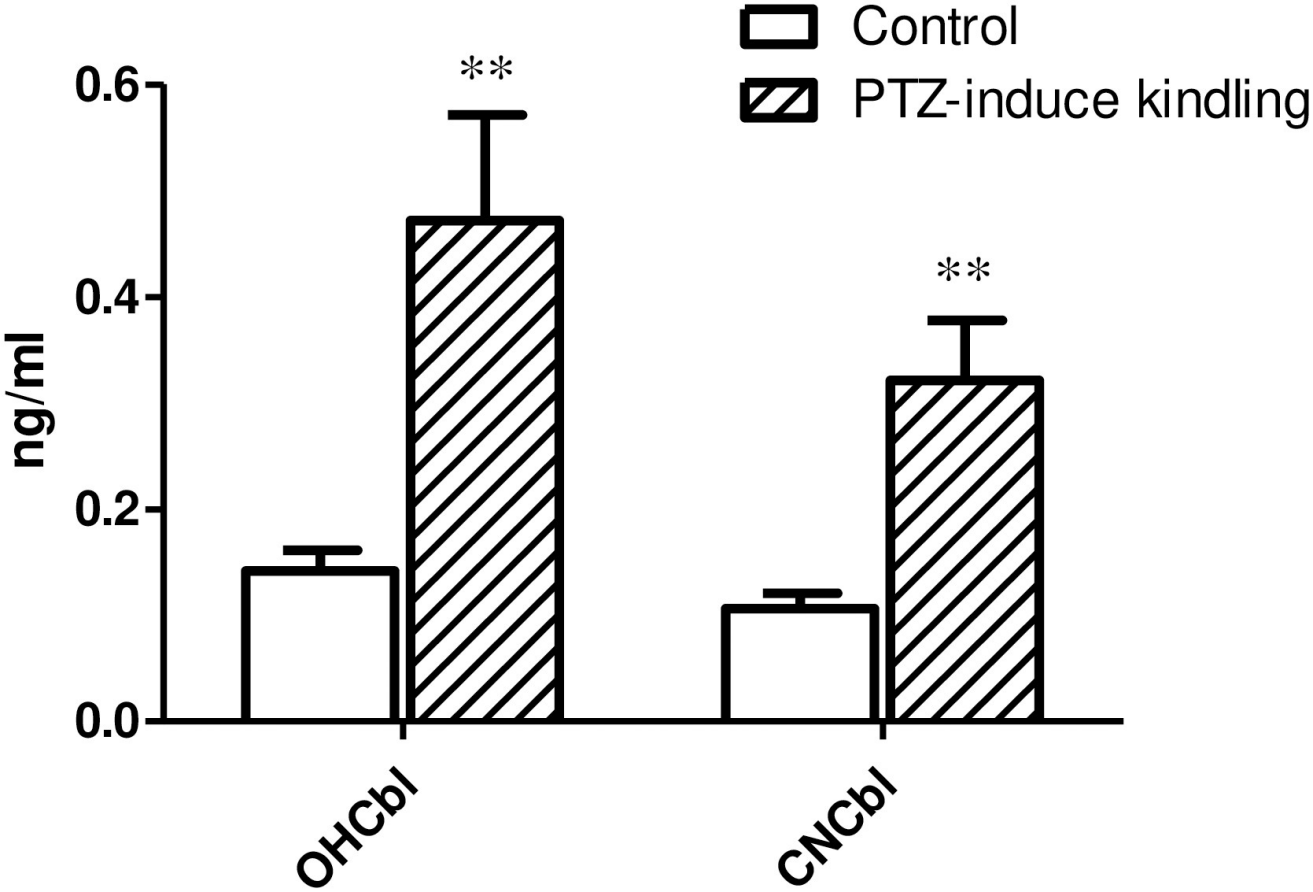
Histograms showing concentrations of OHCbl and CNCbl in PTZ-induced kindling rat model and controls (n = 8). Each bar represents the mean value; variability is shown by standard error of mean (SEM). Asterisks represent significant difference.

After systematic optimization and verification, this established online SPE-HPLC-MS/MS method was utilized to probe the cobalamins levels in our PTZ-induced kindling rat model for the first time. Eight SD rats received PTZ administration once every 48 h for 5 times, while the control rats were injected with saline. After administration, their behaviors was scored according to scale provided by W Fischer and H Kittner [14]. Rats were considered kindled when seizure attack (score ≥ 3) occurred after each PTZ injection. Table 6 lists the scores and cobalamins levels in our rat model. OHCbl and CNCbl were successfully detected and quantified in rat plasma, whereas AdoCbl and MeCbl were below the quantification limit of our method. The MS responses of AdoCbl and MeCbl in plasma were higher than their respective LODs and lower than their LLOQs, which means AdoCbl and MeCbl could be detected but not quantified reliably in rat plasma (data now shown). In histograms of Fig 4, concentrations of OHCbl and CNCbl in PTZ-induced kindling rat model and controls were displayed. Both OHCbl and CNCbl were significantly (*p* < 0.01) up-regulated in PTZ-induced kindling rat model, and they also positively correlated with the score. MeCbl was below the quantification limit in both control and PTZ group. AdoCbl was also below the quantification limit in control group, however, it was detected and quantified in four rats in the PTZ group, reflecting the up-regulation in PTZ-induced kindling model, in line with OHCbl and CNCbl.

**Table 6:** Plasma OHCbl, CNCbl and AdoCbl concentrations (ng/ml) and scores in rat model (8 PTZ-induced kindling rats and 8 controls).

Group	Score	OHCbl	CNCbl	AdoCbl
Control	0	0.112	0.049	BQL
Control	0	0.216	0.092	BQL
Control	0	0.058	0.185	BQL
Control	0	0.099	0.134	BQL
Control	0	0.114	0.107	BQL
Control	0	0.154	0.071	BQL
Control	0	0.192	0.117	BQL
Control	0	0.19	0.094	BQL
PTZ	5	0.778	0.454	0.088
PTZ	5	0.535	0.519	0.059
PTZ	3.5	0.268	0.24	BQL
PTZ	5	0.788	0.453	0.08
PTZ	5	0.782	0.437	0.079
PTZ	4	0.133	0.129	BQL
PTZ	4	0.259	0.153	BQL
PTZ	2.5	0.232	0.184	BQL

BQL: below the quantification limit.

## 4. Conclusions

In this study, we constructed an online SPE-HPLC-MS/MS method for simultaneous analysis of four endogenous cobalamins in rat plasma. Analytes were trapped and enriched on a SPE column and then separated in HPLC-MS system in a short time. Various sample pre-treatment, liquid chromatography and mass spectrometry conditions were tested and optimized. After full validation according to the Food and Drug Administration guidance for industry on bioanalytical methods, this method was successfully applied to detect the four cobalamins in PTZ-induced kindling rat model for the first time.
